# Increasing Ages of *Inga punctata* Tree Soils Facilitate Greater Fungal Community Abundance and Successional Development, and Efficiency of Microbial Organic Carbon Utilization

**DOI:** 10.3390/microorganisms12101996

**Published:** 2024-09-30

**Authors:** William D. Eaton, Debra A. Hamilton

**Affiliations:** 1 Biology Department, Dyson College, Pace University, New York, NY 10038, USA; 2 Department of Environment and Development, University for Peace, El Rodeo de Mora, San José 10701, Costa Rica; 3 Vermont Cooperative Fish and Wildlife Research Unit, Rubenstein School of the Environment and Natural Resources, University of Vermont, Burlington, VT 05405, USA; debrahamiltonmv@gmail.com; 4 Monteverde Institute, Monteverde, Puntarenas 60109, Costa Rica

**Keywords:** fungal decomposition, complex carbon decomposition, *Inga punctata* tree soils, tropical soil fungi, *Inga* reforestation, fungal community complexity, fungal community stability, soil *q*CO_2_, soil respiration, soil biomass C

## Abstract

Leguminous *Inga* trees are thought to enhance soil carbon (C) accumulation following reforestation, through mostly unknown mechanisms. This study amplified soil DNA using the ITS1F and ITS4 primers for PCR and Illumina MiSeq methods to identify fungal taxa, and traditional C analysis methods to evaluate how planted 4-, 8-, and 11-year-old *Inga punctata* trees affected soil fungal community compositions and C utilization patterns compared to old-growth *I. punctata* trees and an adjacent unplanted pasture within the same reforestation zone in Monteverde, Costa Rica. Along the tree age gradient, the planted *I. punctata* trees enhanced the tree soil C capture capacity, as indicated by increased levels of soil biomass C, Respiration, and efficiency of organic C use (with lower *q*CO_2_ values), and development of increasingly more abundant, stable, and successionally developed fungal communities, including those associated with the decomposition of complex organic C compounds. The level and strength of differences coincided with differences in the time of separation between the pasture and tree age or between the different tree ages. Fungal taxa were also identified as potential indicators of the early and late stages of soil recovery. Thus, planting *I. punctata* should be part of future reforestation strategies used in this region of the Monteverde Cloud Forest in Costa Rica.

## 1. Introduction

The conversion of tropical forests for agricultural uses is thought to negatively impact the structure of the soil microbial communities associated with decomposition and efficient conversion of C into biomass C [1,2,3]. This is largely due to a reduction in plant-derived resource inputs into the soils [4,5,6]. Remediation strategies are commonly used to restore the biodiversity of tropical forests [7,8,9]. However, minimal information is available on how these strategies influence the recovery of the damaged microbial communities that are critical for the restoration of the soil C cycle activities and C capture. Such information would help maximize the efficacy of restoration methods used to restore tropical forest soil ecosystem health [10,11,12,13].

The decomposition of organic matter is an important component of soil ecosystem processes, which are damaged during deforestation and require restoration to fully recuperate the soil C cycle activities. Although bacteria are considered important for decomposition during the early stages of soil recovery involving oxidation of less complex, more labile forms of organic C compounds [14,15], they are less efficient than fungi at decomposition of the more complex and recalcitrant forms of organic C that are important for enhancing soil C use efficiency, biomass development, and C capture in soils [16,17,18,19,20,21]. Specifically, fungi have been shown to be more critical than bacteria for decomposition of such complex organic C compounds as lignin, lignocelluloses, polyaromatic compounds, suberins, resins, and others, whose complex biproducts of these oxidations enhance the soil organic C, biomass C, and C use efficiency [17,22,23,24]. As such, changes in the fungal community of complex organic C decomposers should be considered a potential indicator of the later stages of advanced decomposition and soil recovery, as well as the efficacy of remediation activities after forest disturbance.

Tropical forest leguminous trees and their soil microbiomes in either natural or assisted reforestation practices provide the principal pathways for the recovery of the tropical forest soil N and C cycle dynamics following deforestation [25,26,27,28,29,30]. These abilities have led to the common use of these trees in tropical forest restoration strategies [29,30,31]. Members of the genus *Inga* are leguminous trees common throughout the tropical Americas, are ecologically important for enhancing accumulation of both soil N and C, are presumed to influence the soil microbial communities associated with these biogeochemical cycles, and are commonly used in restoration practices [29,32]. However, little is known of how *Inga* spp. influence the soil microbiota, particularly the critical soil fungal community of decomposers. Such information would be valuable for the development of more efficacious restoration strategies using *Inga* to help restore damaged tropical forests in order to enhance the recovery of the soil C cycle dynamics, and to assess the success of such strategies.

In a reforestation site in Monteverde, Costa Rica, *I. punctata* trees were planted over time within a previously intact premontane wet forest area that had been cleared, used for agriculture and pasture, then abandoned. An earlier study [13] showed these tree soils, along a tree age gradient from planted to older forest *I. punctata* trees, had increasing levels of total organic C (TOC) and biomass C, and the abundance and complexity of the lignin-degrading bacteria increased over time. However, missing from this work was a comparison of the tree soil microbial communities to those in the adjacent pasture (which was part of the same the pasture in which the *I. punctata* trees were planted) and consideration of how the trees influenced the soil fungal communities and the efficiency of microbial C utilization, and whether these latter two components could serve as indicators of more advanced soil microbial community succession and soil recovery.

Thus, the purpose of this study was to assess whether the planting of *I. punctata* in abandoned pasture soils enhanced the soil fungal community, converting the soils from C sources to C sinks. To do so, the following objectives were used to further analyze the earlier collected DNA sequences and the soil environmental data for additional information not previously published. We wished to determine (1) if the tree soils of increasing ages of planted *I. punctata* were associated with enhanced microbial efficiency in converting organic C into biomass C (as decreased *q*CO_2_ levels); (2) if the tree soils of increasing ages of planted *I. punctata* were associated with enhancement of the fungal community associated with complex organic C decomposition (CCDec Fungal taxa); (3) if there were changes in the levels of critical fungal taxa associated with the differences in the soil C metrics; and (4) if different fungal taxa became more or less prominent in the soils along the *I. punctata* tree age gradient.

## 2. Materials and Methods

### 2.1. Inga punctata Reforestation Site Description and Soil Sample Collection

Plans to restore abandoned pasture in the Monteverde Cloud Forest Region of Costa Rica have been implemented by the Fundación Conservacionista Costarricense (FCC) and Monteverde Institute (MVI) since 1998 and 2016, respectively (both organizations are in Monteverde, Puntarenas, Costa Rica) These efforts included planting seedling *I. punctata* in three restoration sites within a former farm site, the Finca Rodríguez Ecological Reserve (FRER), located in Monteverde (10°18′55.3″ N, 84°50′29.8″ W) in 2008, 2011, and 2015 in specific plots within the FRER [33]. All plots were on land previously used for coffee, sugar cane, and dairy cattle pasture, then abandoned for four years before each planting. Prior to planting, manual clearing of all grasses was performed, while, after planting, all shrub and natural regeneration in the plots was minimized with weeding of the planted seedling plots, which continued until the year of this study. In August 2019, soil was collected from the base of 6 replicate *I. punctata* trees from each of the 4 age classes: those planted 4, 8, and 11 years prior to sampling (called the Inga 4, 8, and 11 soils) and older (> 50 years old) I. *punctata* trees (called the Old Inga soils) within the FRER. The stands of any of the four tree age classes were 50–300 m apart. The soil collected from any of the planted trees was at least 5 m from any other planted tree in that stand, and the soil collected from the Old Inga trees was at least 15 m apart from other Old Inga trees. For comparative purposes, soil was also collected from 6 pasture plots (25 m × 20 m each) within the FRER (called PAS soils) that were adjacent to the planted *I. punctata* stands, and separated by at least 25 m. The extremely small spatial scale of soil microbial communities is such that a separation of several centimeters to several meters between soil samples is equivalent to an ecosystem-level separation for above-ground communities, and those that are greater than several meters are equivalent to a landscape-level separation of above-ground communities [34,35,36]. Given this, the soils collected for this study represent 6 true soil replicates from each tree age stand and 6 from the pasture.

Soils were collected from beneath the *I. punctata* trees using a combination of methods that have since been characterized by Addison et al. [37], which control for neighbor tree and other environmental effects and ensure all samples are from the same functional location for all trees. This allows for comparison of the potential influence of the aging tree on the specific tree soil fungal community compositions. Specifically, for each of the 6 trees used per tree age class, the 10% distance from the base of the tree to its canopy edge was calculated and considered as the 10% dripline region. Two soil profiles (7.5 cm × 1.25 cm × 15 cm) were collected (from approximately 15 cm depth) at each of the four cardinal points at the 10% dripline distance point for each tree, providing eight soil cores per tree, which were pooled into a single sterile bag per tree. This provided 6 replicate 8-core pooled soil samples from each tree age stand for analysis. Using this method, no other tree canopy radius, shrubs, or regenerating trees were within the 10% dripline zone sampled for each *I. punctata* tree. There were 6 soil cores collected from each of the PAS plots using the same soil profile corer, and using the stratified block systematic plot study design recommended for studies of damaged forest lands (www.epa.gov/sites/default/files/2015-06/documents/g5s-final (accessed on 20 August 2019)). The 6 soil cores from each PAS plot were collected using a pre-determined sample location strategy and placed in a single sterile bag per plot, providing 6 composite, independent replicate soil samples from the pasture region. Prior to all soil collection, any surface leaf litter was carefully removed to expose the upper organic horizon. In addition, all soil samples were aseptically collected by disinfecting all gloves and collection tools with 70% ethanol between trees and between PAS plots. The samples were passed through a sterilized 5 mm sieve under field-moist conditions to homogenize the soil sample prior to analysis. After sampling, following the recommendations of Lucas et al. [38], the field-moist soils were kept in semi-open plastic bags under refrigeration at 4 °C for no more than 1 week prior to analysis, as these authors showed that holding tropical soils under these conditions for up to 12 days did not affect microbial activity results.

### 2.2. Soil Respiration, Biomass C, and qCO_2_

Subsamples of 200 g of field and sieved moist soil per replicate soil sample were analyzed by the Centro Agronómico Tropical de Investigación y Enseñanza (CATIE) in Turrialba, Costa Rica, for all C cycle metrics. The levels of Respiration (as CO_2_) were determined by standard closed-vessel methods, and the microbial C biomass was determined by standard chloroform-fumigation methods [39]. Many studies have discussed how the Metabolic Quotient, or *q*CO_2_, has been used for decades as an indicator of environmental impacts on microbial catabolic activity, the efficiency with which soil microbial communities convert organic C into biomass C, and enhanced soil ecosystem microbial community development, such that, at maturity of microbial diversity in a soil system, there should be a decreased level of Respiration per unit of biomass C generated [40,41,42,43,44,45]. Given this, we used the *q*CO_2_ (the ratio of Respiration to biomass C) with decreasing values to indicate development of a more mature and advanced fungal community of microbes with increased efficiency in converting soil organic C into biomass C. Differences in all mean values were assessed by one-way analysis of variance (ANOVA) followed by Tukey’s HSD or Dunnett’s T3 post hoc test, as appropriate, in SPSS (v.27, Armonk, NY, USA). Prior to ANOVA, Levene’s test and the Shapiro–Wilk test were performed in SPSS to determine homogeneity normality of all the data to support the use of ANOVA.

### 2.3. DNA Extraction, Sequencing, and Bioinformatics

The soil DNA was extracted from three 0.33 g of soil per replicate soil sample using the MoBio PowerSoil DNA Isolation Kit (MO BIO Laboratories Inc., Carlsbad, CA, USA) and the concentration and purity determined using a NanoDrop 1000 spectrophotometer (ThermoFisher Scientific, Waltham, MA, USA) prior to downstream processing. All methods for PCR, DNA sequencing, quality control, Illumina MiSeq sequencing, and bioinformatics used to identify the fungal taxa have been previously explained in detail by McGee et al. [46]. Briefly, the eDNA extracts were amplified by 2-step PCRs targeting the nuclear internal transcribed spacer (ITS) ribosomal RNA gene region for fungi using the ITS1F and ITS4 primers [47], with the resulting amplicons sequenced in Illumina MiSeq runs using a V3 MiSeq sequencing kit (FC-131-1002 and MS-102-3003). The ITS DNA sequences were processed using semi-automated pipelines to generate operational taxonomic units (OTUs) that were taxonomically assigned using the RDP classifier with the UNITE fungal ITS set for fungi [48]. All sequencing data were submitted to the NCBI Gene Expression Omnibus (GEO) repository on 1 August 2019 (Submission: SUB6145149, BioProject: PRJNA559202).

The resulting unique fungal OTUs were categorized into specific fungal taxa, generally at the genus level, and were considered the Total Fungal taxa. We normalized the library size of each taxon for each soil sample by determining the mean percentage of the sequences (MPS) for each taxon, as recommended by Weiss et al. [49], to account for differences in the number of sequence hits between samples. The fungal taxa were categorized as being saprobes associated with decomposition of less complex organic C (SAPDec), root-associated fungal decomposers (RADec), complex organic C decomposers (CCDec), endoparasites (PAR), and arbuscular mycorhyizal fungi (ARM) using the databases www.genome.jp/kegg/ (accessed on 24 January 2020), https://fungidb.org/ (accessed on 25 January 2020), FungalTraits 1.2 ver. 16 [50], the Ribosomal Database Project (RDP) Classifier (https://sourceforge.net/projects/rdp-classifier/) (accessed on 25 January 2020), www.loucalab.com/archive/FAPROTAX/lib/php/index.php?section=Download (accessed on 29 January 2020), and https://www.ars.usda.gov/ (accessed on 28 January 2020) (USA National Fungus Collection) and other literature sources (Supplementary materials). The fungal taxa found to be capable of decomposing complex forms of C and also those that were wood rot fungi are hereafter referred to as the CCDec Fungal taxa. Both the Total Fungal taxa and the CCDec Fungal taxa were used for further analysis. This approach has been recently used by Eaton et al. [4,5,51,52,53,54] and Eaton and Hamilton [13] to link microbial taxa to proposed functional activity within soils from other studies in Costa Rica.

### 2.4. Differences in Fungal Community Compositions

Differences in fungal community composition were assessed by comparing the MPS levels and the Margalef’s richness levels, and by ANOSIM and CAP analyses. The MPS values of two subsets of the Total Fungal community were used to compare mean differences in taxa between the soil groups by the Kruskal–Wallis analysis conducted in SPSS (v. 26): the subset of Total Fungi with MPS values > 1.0% (called the Most Common Taxa) and the representatives from this group identified as CCDec Fungal taxa. The MPS data were 4th root transformed as recommended by Anderson et al. [55] to diminish the influence of extremely abundant or rare taxa and used to determine the Margalef’s richness indices using PRIMER-E v.6 [56]. The richness levels were assessed for differences between soils by ANOVA in SPSS.

Overall taxonomic compositional differences of both the Total Fungal taxa and all CCDec Fungal taxa between the soil habitats were assessed using the ANOSIM routine in the PRIMER-E package v.6 [55] applied to 4th-root-transformed MPS data that were then converted into Bray–Curtis matrices. ANOSIM provides Global and Pairwise R statistics and *p* values for the main and comparative tests that are used to identify differences in mean values between groups. The strength of any community compositional differences between soil groups identified by ANOSIM was determined using the Canonical Analysis of Principal Coordinates (CAP) ordination method [56,57] in the PRIMER-E package v.6 with the add-on PERMANOVA+ applied to the same Bray–Curtis similarity matrices mentioned above. The resulting CAP axis squared canonical correlations (R^2^) provide an approximation of the strength of differences in community compositions between soil samples. Strong differences are indicated by R^2^ values ≥ 0.7, moderate differences by R^2^ = 0.5 to 0.69, weak differences by R^2^ = 0.20 to <0.5, and no differences by R^2^ < 0.20.

### 2.5. Indicators of Fungal Community Successional Development

To examine the data for evidence of successional development in the fungal communities, we compared the MPS levels of the Total Fungal taxa and the CCDec taxa along the gradient from the PAS to Old Inga soils, assessing for increases in the abundance of taxa associated with more successionally advanced soils [5,58,59]. We also analyzed differences in the levels of Margalef’s richness for the different taxa by applying the ANOVA routine to the MPS data levels of each fungal group as successional development in soils often results in decreased taxonomic richness, as with dominant taxa developing over time due to competitive exclusion processes [52,58,59,60,61,62]. Also, the multivariate SIMPER routine was performed on the MPS data in PRIMER-E v.6 [56] to determine the percent contribution of the Total Fungal taxa to their respective total community compositions as another indicator of development of dominant taxa over successional time. From this, the more typical or characteristic taxa for a soil group were determined to be those that provided the greatest percent contribution to the community composition for that soil group.

These successional-related processes ultimately result in an increasing level of taxonomic stability as communities develop into ones demonstrating a more homeostatic relationship with the environmental conditions of the more established and developed soils [63,64,65,66,67,68]. We assessed the taxonomic stability of the Total Fungal and CCDec Fungal communities by calculating the Stability *S* index of the MPS and the richness values as the mean value divided by the standard deviation of that mean, with greater *S* values suggesting greater stability of that community metric [63,64,65,68,69]. The *S* index has previously been used in microbial community studies [13,54,68,69].

### 2.6. Potential Influence of Fungal Taxa on the Carbon Metrics

The distance-based linear model (DistLM) analysis (Primer E v.6 and PERMANOVA+) was implemented to determine the degree to which any of the Most Common Fungal taxa might influence the patterns of the soil biomass C, Respiration, or *q*CO_2._ The MPS data of the Most Common Fungal taxa were used as predictor variables, and the log (x + 1)-transformed C metrics were used as the response variables after converting the transformed C data into Euclidean similarity matrices (Anderson et al. 2008). A stepwise selection process was used, along with an AICc (Akaike’s-Information-Criterion-Corrected) selection criterion and 9999 permutations [55] for the DistLM analysis.

## 3. Results

### 3.1. Differences in C Metrics

There were differences observed in the levels of biomass C, Respiration, and *q*CO_2_ between some of the different soils (Table 1). The biomass C and Respiration were greater in the PAS soils than in the Inga 4 and 8 soils (*p* < 0.0001) and the Inga 11 soils (*p* = 0.0001 and 0.022). The biomass C levels were greater in the Old Inga than in any other soils (*p* range < 0.0001 to 0.031), but the Respiration levels were only somewhat greater in the Old Inga than the Inga 4 and 8 soils (*p* = 0.054 and 0.051), not different between the Old Inga and Inga 11 soils, and lower in the PAS soils (*p* = 0.008). There were no differences in the *q*CO_2_ levels between the PAS, Inga 4, and Inga 8 soils; however, the levels were lower in the Inga 11 and Old Inga soils than in the PAS, Inga 4, and Inga 8 soils (*p* range = 0.0002 to 0.034) and were also lower in the Old Inga soils than in the Inga 11 soils (*p* = 0.051).

### 3.2. Differences in Fungal Community Compositions

**Figure 1:**
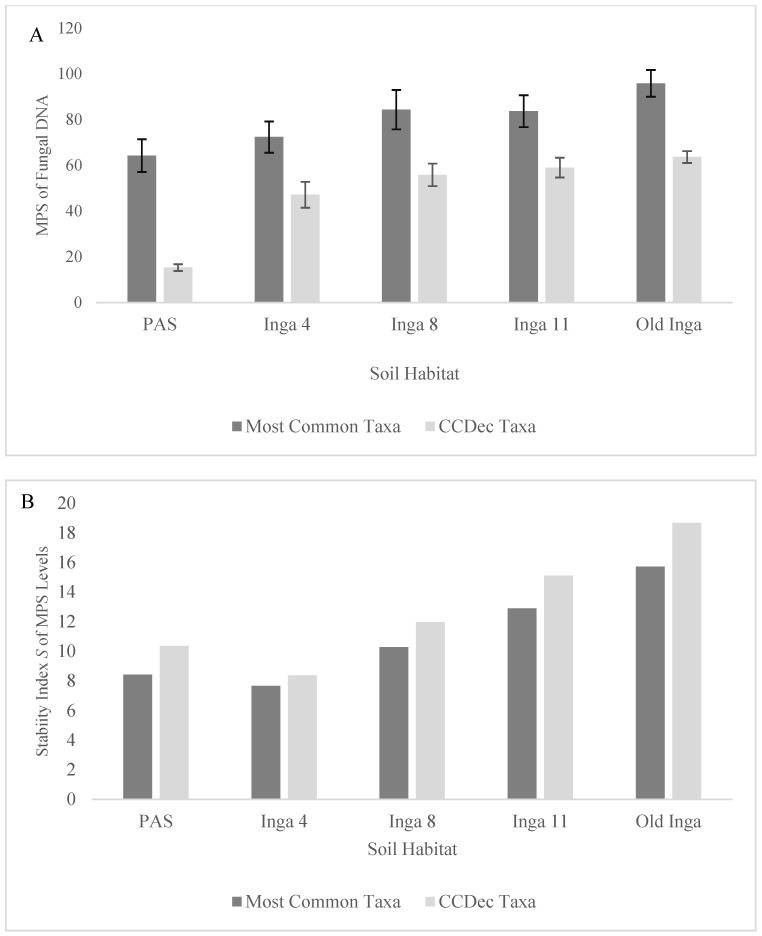
Differences in the MPS of the DNA sequences and the Stability *S* index of the Most Common Fungal taxa (subset of Total Fungi with MPS > 1.0%) and the Complex C Decomposer (CCDec) taxa within soils of 4-year-old, 8-year-old, 11-year-old, and old secondary *I. punctata* trees (Inga 4, 8, and 11 and Old Inga) and an adjacent pasture (PAS) within a reforestation site in Monteverde, Costa Rica. (**A**) shows the MPS of the DNA sequences and (**B**) shows the Stability *S* index for the two communities.

**Figure 2:**
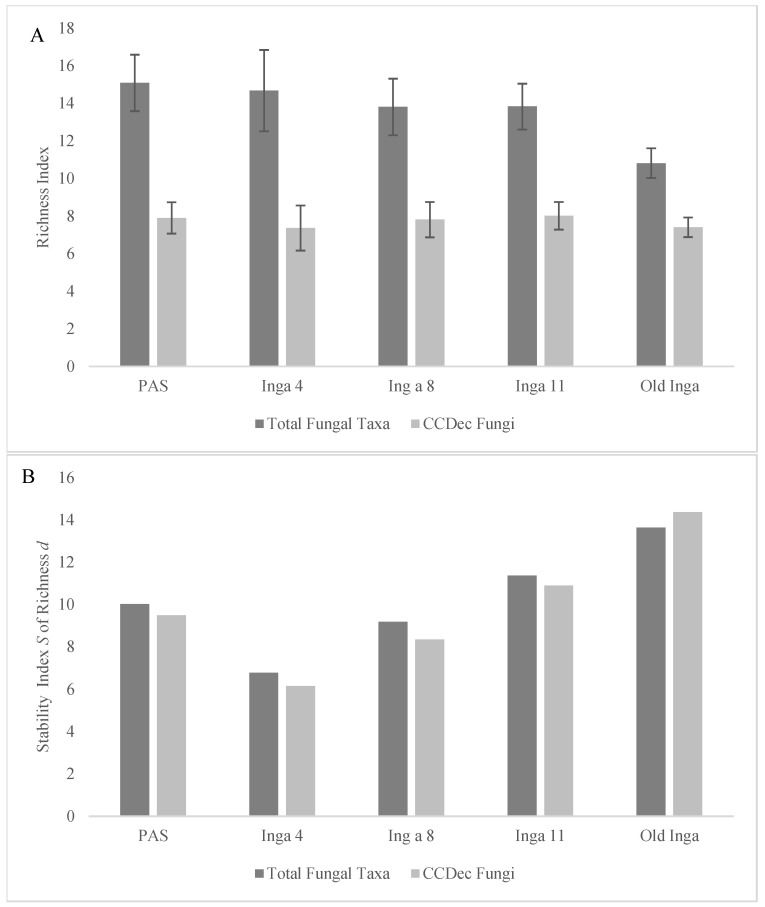
Analysis of the Margalef’s richness index *d* (± standard deviations) and the Stability *S* index of the richness *d* index for the community compositions of the Total Fungi and the Most Common Fungi within soils of 4-year-old, 8-year-old, 11-year-old, and old secondary *I. punctata* trees (Inga 4, 8, and 11 and Old Inga) and an adjacent pasture (PAS) within a reforestation site in Monteverde, Costa Rica. (**A**) shows the Margalef’s richness and (**B**) shows the Stability *S* index for the two communities.

There were 6395 unique fungal OTUs identified in the PAS soils, 2792 in the Inga 4 soils, 3189 in the Inga 8 soils, 6379 in the Inga 11 soils, and 5928 in the Old Inga soils. These unique OTUs were categorized into 685 specific fungal taxa, generally at the genus level, and were considered as the Total Fungal taxa. There were 17 of the Total Fungal taxa from the soil groups with MPS levels of ≥ 1% in one or more of the soil groups, which were called the Most Common Fungal taxa, 11 of which were identified as the CCDec Fungal taxa (Table 2). The MPS levels of the Most Common Fungal taxa (Figure 1A) increased along the tree age gradient as they were lowest in the PAS soils and increased in the Inga 4 soils (64.3% to 72.4%, *p* = 0.052), increased from the Inga 4 to Inga 8 and 11 soils (72.4% to 84.4% and 83.73%, *p* values < 0.02), and increased in the Old Inga soils (95.9%, *p* < 0.025). The MPS levels of the CCDec Fungal taxa (Figure 1A) followed the same pattern. These MPS levels were far lower in the PAS soils (15.3%) than in any other soils (47.16%–65.04%; all *p* values < 0.0001), then increased from the Inga 4 to Inga 8 and 11 soils (45.16% to 55.86% and 59.04%, *p* = 0.017), and increased again to the levels in the Old Inga soils (59.04% to 63.65%, *p* = 0.048). Although there was a trend of decreasing richness of the Total Fungal taxonomic community observed along the tree age gradient (Figure 2A), the Margalef’s richness indices were not different (*p* > 0.237) between the PAS and Inga 4, 8, and 11 soils (d = 13.8 to 15.09). They were the lowest in Old Inga soils (*d* = 10.8; *p* values < 0.002). There were no differences (*p* > 0.284) in the Margalef’s richness indices of the CCDec Fungal community between any of the five soils in this study (*d* = 7.37 to 8.02).

The ANOSIM and CAP assessments showed similar results for the differences in composition of both the Total Fungal and all the CCDec Fungal taxonomic communities (Table 3). These community compositions were separated into three levels of difference that approximately coincided with either time between the PAS and planted tree age or the time between different planted tree ages. There were weak or no differences in either of the two fungal community compositions between the Inga 4 and Inga 8 soils (ANOSIM R = 0.097 and 0.027; CAP R^2^ = 0.044 and to 0.019) and the Inga 8 and Inga 11 soils (ANOSIM R = 0.323 to 0.386; CAP R^2^ = 0.449 to 0.233). There were moderate differences in the two fungal community compositions between the PAS and Inga 4, PAS and Inga 8, and the Inga 8 and Old Inga soils (ANOSIM R = 0.360 to 0.503; all CAP R^2^ values = 0.568). The greatest differences in both fungal community compositions were found between the PAS and Inga 11, PAS and Old Inga, the Inga 4 and Inga 11, and Inga 4 and Old Inga soils, which demonstrated strong differences between the soils (ANOSIM R = 0.564 to 0.669; CAP R^2^ = 0.745 to 0.791).

### 3.3. Indicators of Fungal Community Successional Development

Soil ecosystems in older established forests, recovering from damage, or undergoing restoration often demonstrate several community compositional patterns linked to microbial community successional development, which were assessed in this study. During succession in soil communities, there is often an increase in the abundance of critical microbiota associated with the decomposition of more complex organic C compounds [5,70,71]. The indicators of community successional development used in this study were the MPS and richness levels of the fungal groups, the Stability *S* index of both metrics for these two groups, and the presence of taxa typical of the different soils. As mentioned above, there were significant increases in the MPS of both the Most Common Fungal taxa and the CCD Fungal taxa along the tree age gradient from the PAS to the Old Inga tree soils (Table 2, Figure 1A). As well, also as mentioned above, there were no significant differences in the richness of the CCDec taxa across the soils, and the richness of the Total Fungal taxa was not different between the PAS and Inga 11 soils but was less in the Old Inga soils (Figure 2A). However, the Stability *S* index values for the MPS values for both the Most Common Fungal and the CCDec Fungal taxonomic communities followed similar patterns (Figure 1B), as they decreased from that in the PAS (8.4 and 10.4) to the Inga 4 soils (7.6 and 8.4), then increased in the Inga 8 soils (10.3 and 11.9), increased in the Inga 11 soils (12.9 and 15.1), and increased again in the Old Inga soils (15.7 and 18.7). Interestingly, the Stability *S* indices of the MPS and the Margalef’s richness values for both the Most Common Fungal and the CCDec Fungal communities showed the same pattern (Figure 2B). These *S* values decreased between the PAS (10.0 and 9.5, respectively) and the Inga 4 soils (6.8 and 6.2, respectively), then increased each year in the Inga 8 soils (9.2 and 8.3, respectively), Inga 11 soils (11.4 and 10.9, respectively), and the Old Inga soils (13.6 and 14.4, respectively).

There were several clear patterns of differences in taxa that could be considered as typical of the different soils based on their percent contribution to the Total Fungal community composition and the MPS levels (Table 2 and Table 4). The CCDec taxa *Saitozyma* and *Apiotricum* demonstrated opposite patterns in the Inga 4 and 8 and the Inga 11 and Old Inga soils that were also different from that in the PAS soils (Table 2 and Table 4). In the Inga 4 and 8 soils, *Saitozyma* contributed 16.48% and 15.35% to the Total Fungal community composition, while contributing 10.95% and 10.24% to the community in the Inga 11 and Old Inga soils and only 0.17% in the PAS soils (0.17%). Consistent with this, the MPS of *Saitozyma* was 29.42% and 28.89% in the Inga 4 and 8 soils, and <9% in all other soils (*p* < 0.042). In contrast, *Apiotrichum* contributed 22.38% and 24.53% to the Total Fungal community composition in the Inga 11 and Old Inga soils, 0.06% and 6.99% in the Inga 4 and 8 soils, and 9.48% in the PAS soils, while having MPS values of 43.61% and 41.96% in the Inga 11 and Old Inga soils and less than 6.5% in all other soils (*p* < 0.016).

The CCDec taxon *Tremella* appeared to be more critical in the Old Inga soils as there it contributed 10.23% to the Total Fungal community composition but less than 0.2% in all other soils. As well, it had an MPS value of 8.21 in the Old Inga soils and less than 1.6% in all other soils (*p* < 0.035). The SAPDec fungus *Starmerella* appeared to be somewhat more important in the Inga 11 and Old Inga soils, where it contributed 10.45% and 14.77% to the Total Fungal community composition but contributed less than 6% in all other soils. It also had MPS levels of 8.13% and 11.44% in the Inga 11 and Old Inga soils and less than 3% in all other soils (*p* < 0.051). Members of the CCDec Fungal family Sordariaceae contributed 9.39% and 9.85% to the community composition in the Inga 4 and Inga 8 soils, and less than 5.84% for all others, and also had MPS values of 7.98% and 11.6% in these soils compared to less than 1.5% for all others (*p* < 0.024).

There were three taxa that showed clear differences in contribution to the PAS soils compared to the other soils. The RADec taxon *Archaeorhyzomyces* contributed 18.22% to the community composition in the PAS soils and less than 11.5% in all others. It also had an MPS level of 32.11% in the PAS soils and less than 5.5% in all other soils (*p* < 0.015). Additionally, *Rozella*, the endoparasite (PAR) of fungi and oomycetes, contributed 13.69% to the community composition in the PAS soils and less than 5.5% in all other soils, with an MPS of 7.97% in the PAS soils and less than 2% in all others (*p* < 0.037). The arbuscular mycorrhizal fungal group Glomeromycota was found in all the soils, but the PAS soils had the lowest percent contribution of this group (9.45% vs. > 16.2% in all others) and the lowest MPS values (4.8% vs. >12.7% in all others, *p* < 0.046) compared to the other soils.

Lastly, the overall % contribution of the total CCDec taxa to the Total Fungal taxonomic community increased from that in the PAS and in each soil sample along the tree age gradient. The percent contribution of the CCDec taxa to the total community composition increased from 25.11% in the PAS soils to 42.41% in the Inga 4 soils, to 47.026% in the Inga 8 soils, to 55.13% in the Inga 11 soils, and to 58.76% in the Old Inga soils. As mentioned above, this occurred with the significant increases (*p* values from <0.0001 to 0.048) in total CCDec MPS levels from the PAS soils (15.3%) to the Inga 4 soils (47.16%), to the Inga 8 and Inga 11 soils (55.86% and 59.04%, respectively), and to the Old Inga soils (63.65%).

### 3.4. Potential Influence of Fungal Taxa on the Carbon Metrics

The DistLM analysis (Table 5) showed that *Saitozyma* and *Apiotrichum* were the most probable fungi influencing the patterns of the biomass C, Respiration, and *q*CO_2_ as these two taxa explained 43.97% of the combined patterns of the biomass C, Respiration, and *q*CO_2_ metrics. These two taxa also explained 54.82% and 41.99% of the individual patterns of the Respiration and *q*CO_2_ values, respectively, while *Saitozyma* was the only taxon that explained the individual patterns of biomass C values, as it explained 48.67% of the patterns of the biomass C values.

## 4. Discussion

The results from this project indicate that the Total Fungal community and its two subset communities of the Most Common Fungal and CCDec Fungal taxa are becoming more successionally advanced over time in the *I. punctata* tree soils compared to the pasture soils, which is occurring concurrently with increases in soil biomass C and Respiration and enhanced efficiency of converting organic C into biomass C (lower *q*CO_2_). This indicates that planting *I. punctata* enhances the tree soil fungal microbiome and associated C cycle activities, resulting in the soil becoming more of a C sink, as opposed to being more of a C source, as is the case with the pasture soils (high Respiration rate and high *q*CO_2_). There are several specific lines of evidence for this from the study. Specifically, coincident with the increasing levels of biomass C and Respiration and decreasing *q*CO_2_ occurring along the tree age gradient, the ANOSIM and CAP results showed the Total Fungal and CCDec community compositions were different between the different tree soils, with both the level and strength of difference coinciding with the difference in time of separation between the PAS and planted trees or with the separation of the ages of the planted trees themselves. Moreover, these compositional differences were associated with increasing levels of the Stability *S* index of the MPS and the richness of both fungal groups along the tree age gradient. These data suggest the taxa within these fungal groups are becoming more dominant and stable over time in the soils, which would be expected of microbial communities in soils undergoing succession or recovery from damage [5,58,62,67,68,69].

It is known that different microbial taxa will become more typical of a soil habitat undergoing succession or recovery from damage as competitive exclusion occurring within the soils facilitates the selection of taxa better fit for the different niches that develop over time [58,61,62]. The current study suggests certain taxa are more indicative of certain stages of soil microbiotic successional development. Specifically, the CCDec taxa *Saitozyma* and Sordariaceae were more typical of the Inga 4 and Inga 8 soil communities, while the CCDec taxon *Apiotrichum* and the plant saprobe *Starmerella* were more typical of the Inga 11 and Old Inga soil communities, and the CCDec taxon *Tremella* was more typical of the Old Inga tree soil. The value of this may be that the greater MPS levels of *Saitozyma* and Sordariaceae are indicators of the early stages of soil ecosystem recovery after damage, while the greater MPS levels of *Apiotrichum* and *Starmerella* are indicators of the later stages of complex organic C compound decomposition and advanced soil ecosystem recovery after damage. This is supported by the DistLM results, which indicate that *Saitozyma* and *Apiotrichum* were the greatest predictors of the differences found in the biomass C, Respiration, and *q*CO_2_, and suggests that they are important for differentially decomposing complex organic C materials in the early and later stages of soil recovery post-tree planting.

The PAS soil also had several taxa that may serve as being more characteristic of these soils as compared to the tree soils. *Archaeorhizomyces* and *Rozella* contents were greater in the PAS soils than in the other soils. Consistent with this, *Archaeorhizomyces* and *Rozella* have both been found to be more common in pastures than forest soils [72,73,74,75,76,77]. The greater presence of *Archaeorhizomyces* in the pasture soils may be because it is a non-mycorrhizal root-associated saprotroph that should thrive in the thick root mass of pasture soils (Pinto-Figueroa et al. 2019; Rosling et al. 2011). However, it is not evident why the endoparasite *Rozella* would be dominant in the pasture soils. The levels of the arbuscular mycorrhizal (ARM) Glomeromycota were much lower in the PAS soils than in any of the other soils, which supports the recent review that found Glomeromycota taxa to be somewhat more common in tropical and subtropical forest soils than in adjacent pasture soils [78], which may be due to the greater abundance and diversity of woody plants as ARM hosts in the forested areas. This point needs further work to clarify what could be occurring within the Glomeromycota community in these different soil groups. Nonetheless, in addition to increases in levels of *Saitozyma* and Sordariaceae and increases in levels of *Apiotrichum* and *Starmerella* serving as indicators of the early and then the later stages of recovery, respectively, it may also be that higher levels of Glomeromycota are an indicator of soils undergoing recovery.

The increase in the CCDec Fungal community MPS, Stability, successional development, and percent contribution to the Total Fungal community composition along the tree age gradient suggests the importance of the fungal CCDec activity within all tree soil age groups. Further, it appears that, about 8–11 years after the planting of *I. punctata*, this taxonomic community reaches a level of homeostasis of taxonomic distribution within the soils that results in an increase in community compositional stability. This is also consistent with the pattern of functional redundancy that may be occurring in the soils associated with CCDec taxa, as these fungal taxa possessing similar metabolic capabilities experience community compositional changes while living within similar niches that are undergoing successional changes over time [58,77,79]. Though more common in bacterial communities, functional redundancy has been shown to occur in fungal communities, although the mechanisms are not clear [80,81,82]. Regardless, it appears that the fungal CCDec community has undergone a rapid successional development in the soils post-tree planting, which warrants further study as it suggests this group may be an indicator of soil recovery.

## 5. Conclusions

The present study showed that, following the deforestation of a tropical Cloud Forest area and its conversion to agricultural use, planting *I. punctata* in the abandoned pastures appears to facilitate (1) the successional development of soil fungal community taxa that are experiencing an increase in MPS levels and Stability of MPS levels and taxonomic richness of the fungal communities; (2) an increase in the abundance and importance (as % contribution) of the CCDec Fungal taxa that are critical for complex organic C decomposition in the soil; and (3) the presence of certain fungal taxa that are possibly characteristic of earlier and later stages of soil recovery. All of these changes were associated with increases in the soil’s capacity to serve as a C sink, which was indicated by the increase in soil biomass C development, Respiration, and efficiency of conversion of organic C to biomass (*q*CO_2_).

This information could be fundamental to understanding the role of tropical leguminous trees in enhancing the soil fungal community and soil C capture and recovery post-disturbance. This is especially timely now as some have questioned whether tropical leguminous trees have positive [83] or negative [84,85] influences on tree regrowth patterns in reforested tropical areas. It is likely that there are many other co-varying biotic components, including the influence these trees have on the soil fungal microbiomes, that are important drivers of tropical forest regeneration pathways [51,86]. Thus, a more comprehensive understanding of the role that dominant leguminous trees such as *I. punctata* have on the recovery of the tropical forest soil fungal communities critical to the C cycle is needed. This information could help in the development of more efficacious tropical reforestation and forest management plans and provide more insight into what components are driving individual tree species’ regeneration pathways during restoration [12,51,54,82]. In any case, this current study indicates that planting *I. punctata* should be part of future restoration and reforestation strategies used in this Cloud Forest Region of Costa Rica to repair the soil ecosystem decomposition activities that are critical for recuperation of healthy C cycle activities and overall forest recovery.

## Figures and Tables

**Table 1:** Mean values (±standard deviations) of the biomass C, Respiration (Resp), and *q*CO_2_ from soils of *Inga punctata* trees planted 4, 8, and 11 years before sampling, Old Inga trees (>50 years old), and an adjacent pasture (PAS) within a restoration area in Monteverde, Costa Rica (**A**). Results of ANOVA tests for differences between the means of these metrics between the different soils (**B**).

**(A)**			
**Habitat**	**Biomass C (µgCO_2_/g Soil)**	**Resp (µgCO_2_/g Soil)**	***q*CO_2_ (Resp/Biomass C)**
PAS	806.32 ± 64.57	261. 04 ± 22.58	0.33 ± 0.06
Inga 4	547.50 ± 31.05	180.67 ± 19.49	0.33 ± 0.04
Inga 8	540.67 ± 17.13	181.33 ± 14.17	0.34 ± 0.03
Inga 11	725.33 ± 58.29	201.65 ± 28.90	0.28 ± 0.03
Old Inga	934.83 ± 70.06	209.83 ± 30.47	0.22 ± 0.04
**(B)**			
**Comparisons**	***p* Values for Comparisons of Means**		
	**Biomass C**	**Respiration**	***q*CO_2_**
PAS to Inga 4	<0.0001	<0.0001	1
PAS to Inga 8	<0.0001	<0.0001	1
PAS to Inga 11	0.0001	0.022	0.034
PAS to Old Inga	0.031	0.008	0.006
Old Inga to Inga 4	<0.0001	0.054	0.0002
Old Inga to Inga 8	<0.0001	0.051	0.0005
Old Inga to Inga 11	0.0002	0.644	0.015
Inga 11 to Inga 4	0.0001	0.145	0.006
Inga 11 to Inga 8	0.0001	0.143	0.033
Inga 8 to Inga 4	0.6472	0.942	0.635

**Table 2:** The 17 Total Fungal taxa with MPS > 1%, considered the Most Common Fungal taxa, within soils of 4-year-old, 8-year-old, 11-year-old, and old secondary *I. punctata* trees (Inga 4, 8, and 11 and Old Inga) and an adjacent pasture (PAS), and the CCDec within a reforestation site in Monteverde, Costa Rica. (Key To Functions: SAPDec: saprobes that decompose simpler forms of organic C; RADec: root-associated decomposers; CCDec: complex organic C decomposers; PAR: endoparasite; ARM: arbuscular mycorrhizal fungi.

Taxa	Function	MPS PAS	MPS Inga 4	MPS Inga 8	MPS Inga 11	MPS Old Inga
*Apiotrichum*	CCDec	5.37%	0.19%	6.16%	43.61%	41.96%
*Archaeorhizomyces*	RADec	32.11%	3.74%	5.31%	2.02%	3.53%
*Chaetomium*	CCDec	0.29%	1.73%	0.99%	0.3%	0.57%
*Dipodascus*	CCDec	4.21%	0%	0.56%	2.02%	0.54%
*Geotrichum*	CCDec	0.18%	0%	0.07%	1.28%	0.22%
Glomeromycota	ARM	4.8%	19.29%	18.41%	12.65%	15.93%
*Leohumicola*	SAPDec	2.46%	0.05%	0.17%	0%	0.38%
*Lipomyces*	CCDec	0%	0.23%	1.54%	1.01%	0.99%
*Mortierella*	CCDec	0.37%	1.15%	0.38%	0.02%	0.45%
*Phialocephala*	CCDec	1.87%	3.56%	2.23%	1.84%	0.89%
Pleosporales	SAPDec	1.64%	0.46%	0.38%	0.28%	0.2%
*Pyrenochaetopsis*	CCDec	0.79%	0.23%	1.94%	0.77%	0%
*Rozella*	PAR	7.97%	0.76%	1.92%	1.61%	0.84%
*Saitozyma*	CCDec	0.74%	29.42%	28.89%	7.68%	8.77%
Sordariaceae	CCDec	1.44%	7.98%	11.6%	0.29%	1.05%
*Starmerella*	SAPDec	0%	2.95%	2.35%	8.13%	11.44%
*Tremella*	CCDec	0.04%	0.67%	1.5%	0.22%	8.21%
**MPS of CCDec Taxa**		15.30%	47.16%	55.86%	59.04%	63.65%

**Table 3:** Differences in the fungal community compositions from soils of *Inga punctata* trees planted 4, 8, and 11 years before sampling, Old Inga trees (>50 years old), and an adjacent pasture (PAS) within a restoration area in Monteverde, Costa Rica. (**a**) The results of ANOSIM and Canonical Analysis of Principal Coordinates (CAP) methods applied to the MPS data of the Total Fungal taxa. (**b**) The results of ANOSIM and Canonical Analysis of Principal Coordinates (CAP) methods applied to the MPS data of the CCDec Fungal taxa.

**(a)**					
**ANOSIM of Total Fungal Taxa**			**CAP of Total Fungal Taxa**		
**Global R = 0.404**			**CAP Model *p* Value = 0.0024**		
**Global *p* Value 0.0001**					
**Pairwise Groups**	**R Statistic**	***p* Values**	**Comparisons**	**R^2^ Value**	**Strength of Diff.**
PAS and Inga 4	0.369	0.015	PAS and Inga 4	0.568	moderate
PAS and Inga 8	0.439	0.002	PAS and Inga 8	0.568	moderate
PAS and Inga 11	0.576	0.002	PAS and Inga 11	0.791	strong
PAS and Old Inga	0.564	0.009	PAS and Old Inga	0.791	strong
Inga 4 and Inga 8	0.097	0.182	Inga 4 and Inga 8	0.044	no difference
Inga 4 and Inga 11	0.669	0.002	Inga 4 and Inga 11	0.791	strong
Inga 4 and Old Inga	0.564	0.016	Inga 4 and Old Inga	0.791	strong
Inga 8 and Inga 11	0.323	0.019	Inga 8 and Inga 11	0.449	weak
Inga 8 and Old Inga	0.503	0.053	Inga 8 and Old Inga	0.568	moderate
Inga 11 and Old Inga	0.201	0.037	Inga 11 and Old Inga	0.044	no difference
**(b)**					
**ANOSIM of CCDec Fungal Taxa**			**CAP of CCDec Fungal Taxa**		
**Global R = 0.473**			**CAP model *p* Value = 0.0006**		
**Global *p* Value 0.0001**					
**Pairwise Groups**	**R Value**	***p* Value**	**Pairwise Groups**	**R^2^ Value**	**Strength of Diff.**
PAS and Inga 4	0.379	0.019	PAS and Inga 4	0.582	moderate
PAS and Inga 8	0.533	0.004	PAS and Inga 8	0.687	moderate
PAS and Inga 11	0.654	0.002	PAS and Inga 11	0.745	strong
PAS and Old Inga	0.616	0.006	PAS and Old Inga	0.745	strong
Inga 4 and Inga 8	0.027	0.359	Inga 4 and Inga 8	0.019	no difference
Inga 4 and Inga 11	0.853	0.002	Inga 4 and Inga 11	0.745	strong
Inga 4 and Old Inga	0.648	0.024	Inga 4 and Old Inga	0.745	strong
Inga 8 and Inga 11	0.368	0.039	Inga 8 and Inga 11	0.233	weak
Inga 8 and Old Inga	0.607	0.002	Inga 8 and Old Inga	0.582	moderate
Inga 11 and Old Inga	0.320	0.017	Inga 11 and Old Inga	0.019	no difference

**Table 4:** The percent (%) contribution of the top 10 fungal taxa with their functions and the CCDec Fungal taxa (% Contrib CCDec) to the Total Fungal community composition within soils of 4-, 8-, 11-year-old, and old *I. punctata* trees (Inga 4, 8, and 11 and Old Inga) and adjacent pasture (PAS) within a reforestation site in Monteverde, Costa Rica.

**% Contribution of Fungal Taxa to the Total Fungal Community Composition in PAS Soils**			**% Contribution of Fungal Taxa to the Total Fungal Community Composition in Inga 4 Soils**		
**Species**	**% Contribution**	**Function**	**Species**	**% Contribution**	**Function**
*Archaeorhizomyces*	18.22	RADec	Glomeromycota	22.63	ARM
*Rozella*	13.69	PAR	*Saitozyma*	16.48	CCDec
*Apiotrichum*	9.48	CCDec	*Phialocephala*	9.84	RACCDec
Glomeromycota	9.45	ARM	Sordariaceae	9.39	CCDec
*Dipodascus*	7.32	CCDEC	*Archaeorhizomyces*	6.25	RADec
*Leohumicola*	3.91	SAPDec	*Starmerella*	5.96	SAPDec
Pleosporales	3.58	SAPDec	*Chaetomium*	3.69	CCDec
Sordariaceae	3.48	CCDec	*Mortierella*	3.01	CCDec
*Phialocephala*	2.98	CCDec	*Rozella*	2.61	PAR
*Pyrenochaetopsis*	1.85	CCDec	Pleosporales	1.78	SAPDec
**Total % Contr CCDec**	**25.11**		**Total % Contr CCDec**	**42.41**	
**% Contribution of Fungal Taxa to the Total Fungal Community Composition In Inga 8 Soils**			**% Contribution of Fungal Taxa to the Total Fungal Community Composition in Inga 11 Soils**		
**Species**	**% Contribution**	**Function**	**Species**	**% Contribution**	**Function**
Glomeromycota	18.65	ARM	*Apiotrichum*	22.38	CCDec
*Saitozyma*	15.35	CCDec	Glomeromycota	16.28	ARM
*Archaeorhizomyces*	11.12	RADec	*Saitozyma*	10.95	CCDec
Sordariaceae	9.85	CCDec	*Starmerella*	10.45	SAPDec
*Apiotrichum*	6.99	CCDec	*Dipodasus*	8.16	CCDec
*Phialocephela*	6.06	CCDec	*Archaeorhizomyces*	6.24	RADec
*Starmerella*	5.18	SAPDec	*Phialocephala*	5.16	CCDec
*Rozella*	5.11	PAR	*Rozella*	4.82	PAR
*Pyrenochaetopsis*	4.63	CCDec	*Geotrichum*	4.35	CCDec
*Lipomyces*	4.14	CCDec	*Lipomyces*	4.13	CCDec
**Total % Contr CCDec**	**47.02**		**Total % Contr CCDec**	**55.13**	
**% Contribution of Fungal Taxa to the Total Fungal Community Composition in Old Inga Soils**					
**Species**	**% Contribution**	**Function**			
*Apiotrichum*	25.53	CCDec	**Key To Functions:** RADec: root-associated decomposers SAPDec: saprobic simple C decomposers CCDec: complex C decomposers PAR: endoparasites		
Glomeromycota	17.62	ARM			
*Starmerella*	14.77	SAPDec			
*Saitozyma*	10.24	CCDec			
*Tremella*	10.23	CCDec			
*Archaeorhizomyces*	8.19	RADec			
Sordariaceae	5.84	CCDec			
*Rozella*	4.24	PAR			
*Lipomyces*	3.85	CCDec			
*Phialocephala*	3.07	CCDec			
**Total % Contr CCDec**	**58.76**				

**Table 5:** Distance-based linear modeling (DistLM) sequential tests showing the fungal taxa with the greatest effects on the variation in the biomass C, Respiration, and *q*CO_2_ levels collectively from all soils within a reforestation site in Monteverde, Costa Rica.

Effects on All C metrics	AICc	Pseudo-F	*p* value	Variation	Cumulative Variation
*Saitozyma*	24.793	11.03	0.0001	29.79%	29.79%
*Apiotrichum*	20.996	6.3272	0.0093	14.18%	43.97%
Effects on Biomass C	AICc	Pseudo-F	*p* value	Variation	Cumulative Variation
*Saitozyma*	16.549	24.656	0.0003	48.67%	48.67%
Effects on Respiration	AICc	Pseudo-F	*p* value	Variation	Cumulative Variation
*Saitozyma*	16.362	17.637	0.0003	40.42%	40.42%
*Apiotrichum*	11.137	7.9661	0.0096	14.40%	54.82%
Effects on *q*CO_2_	AICc	Pseudo-F	*p* value	Variation	Cumulative Variation
*Apiotrichum*	7.7003	9.1392	0.0055	26.00%	26.00%
*Saitozyma*	3.4048	6.8898	0.0153	15.99%	41.99%

## Data Availability

All DNA sequencing data were submitted to the NCBI Gene Expression Omnibus (GEO) repository on 1 August 2019 (Submission: SUB6145149, BioProject: PRJNA559202). All other data are available upon request.

## Supplementary Materials

The following supporting information can be downloaded at https://www.mdpi.com/article/10.3390/microorganisms12101996/s1. References [87,88,89,90,91,92,93,94,95,96,97,98,99,100,101,102,103,104,105,106,107,108,109,110,111] used to identify fungal genera with the capacity to degrade complex organic C and/or act as wood rot fungi, found within the soils of this study can be found in Supplementary Materials.
