# Supplementary material for: Increasing Ages of Inga punctata Tree Soils Facilitate Greater Fungal Community Abundance and Successional Development, and Efficiency of Microbial Organic Carbon Utilization
Source: Microorganisms. 2024 Sep 30;12(10):1996. doi: 10.3390/microorganisms12101996 (PMC11509470; doi:10.3390/microorganisms12101996)
Supplement: Supplementary file 1 [file microorganisms-12-01996-s001.zip › microorganisms-3190655-supplementary.pdf]

Supplementary Material. References used to identify fungal genera with the capacity to degrade complex organic C and/or act as wood rot fungi, found within the soils of the current study.

Abdel-Azeem, A.M.; Gherbawy, Y.A.; Sabry, A.M. Enzyme profiles and genotyping of Chaetomium globosum isolates from various substrates. *Plant Biosyst.* **2016**, *150*, 420–428.

Abdel-Azeem, A.M.; Abu-Elsaoud, A.M.; Abo Nahas, H.H.; Abdel-Azeem, M.A.; Balbool, B.A.; Mousa, M.K.; Ali, N.H.; Darwish, A.M.G. Biodiversity and industrial applications of genus Chaetomium. In *Industrially Important Fungi for Sustainable Development*; Biodiversity and Ecological Perspectives; Springer International Publishing: Cham, Switzerland, 2021; Volume 1. https://doi.org/10.1007/978-3-030-67561-5_5.

Alimadadi, N.; Soudi, M.R.; Wang SAn QiWang, M.; Talebpour, Z.; Yan Bai, F. Starmerella orientalis f.a., sp. Nov., an ascomycetous yeast species isolated from flowers. *Int. J. Syst. Evol. Microbiol*. **2016**, *66*, 1476–1481. https://doi.org/10.1099/ijsem.0.000905

Aliyu, H.; Gorte, O.; Neumann, A.; Ochsenreither, K. Global Transcriptome Profile of the Oleaginous Yeast Saitozyma podzolica DSM 27192 Cultivated in Glucose and Xylose. *J. Fungi* **2021**, *7*, 758. https:// doi.org/10.3390/jof7090758

Bilański, P.; Grad, B.; Kowalski, T. Pyrenochaeta fraxinina as colonizer of ash and sycamore petioles, its morphology, ecology, and phylogenetic connections. *Mycol. Prog.* **2022**, *21*, 74. https://doi.org/10.1007/s11557-022-01827-8

Čadež, N.; Drumonde-Neves, J.; Sipiczki, M.; Dlauchy, D.; Lima, T.; Pais, C.; Schuller, D.; Franco-Duarte, R.; Lachance, M.A.; Péter, G. *Starmerella vitis* fa, sp. nov., a yeast species isolated from flowers and grapes. *Antonie Leeuwenhoek* **2020**, *113*, 1289–1298. https://doi.org/10.1007/s10482-020-01438-x

Cai, L.; Jeewon, R.; Hyde, K.D. Phylogenetic investigations of Sordariaceae based on multiple gene sequences and morphology. *Mycol. Res.* **2006**, *110*, 137–150

Dix, N.J.; Webster, J. *Fungal Ecology*; Chapman and Hall: London, UK, 1995; 556p. https://doi.org/10.1007/978-94-011-0693-1

Gonçalves, P.; Gonçalves, C.; Brito, P.H.; Sampaio, J.P. The Wickerhamiella/Starmerella clade-A treasure trove for the study of the evolution of yeast metabolism. *Yeast* **2020**, *37*, 313–320. https://doi.org/10.1002/yea.3463

Gong, Z.; Wang, Q.; Shen, H.; Hu, C.; Jin, G.; Zhao, Z.K. Co-fermentation of cellobiose and xylose by Lipomyces starkeyi for lipid production. *Bioresour. Technol.* **2012**, *117*, 20–24.

Holland, H.L. Investigation of the carbon- and sulfur-oxidizing capabilities of microorganisms by active-site modeling. In *Advances in Applied Microbiology*; Neidleman, S.L., Laskin, A.I., Eds.; Academic Press: Cambridge, MA, USA, 1997; Volume 44, pp. 125–165, ISSN 0065-2164, ISBN 9780120026449. https://doi.org/10.1016/S0065-2164(08)70461-1.

Kedves, O.; Kocsubé, S.; Bata, T.; Andersson, M.A.; Salo, J.M.; Mikkola, R.; Salonen, H.; Szűcs, A.; Kedves, A.; Kónya, Z.; et al. Chaetomium and chaetomium-like species from European indoor environments include *Dichotomopilus finlandicus* sp. nov. *Pathogens* **2021**, *10*, 1133. https://doi.org/10.3390/pathogens10091133

Ladevèze, S.; Haon, M.; Villares ACathala, B.; Grisel, S. The yeast Geotrichum candidum encodes functional lytic polysaccharide monooxygenases. *Biotechnol. Biofuels* **2017**, *10*, 215. https://doi.org/10.1186/s13068-017-0903-0

Mandyam, K.; Jumpponen, A. Seeking the elusive function of the root-colonising dark septate endophytic fungi. *Stud. Mycol.* **2005**, *53*, 173–189. https://doi.org/10. 3114/sim.53.1.173

Matos, I.T.S.R.; Cassa-Barbosa, L.A.; Costa-Neto, P.Q.; Astolfi-Filho, S. Cultivation of Trichosporon mycotoxinivorans in sugarcane bagasse hemicellulosic hydrolyzate. *Electron. J. Biotechnol.* **2012**, *15*, 12–18.

Menkis, A.; Urbina, H.; James, T.Y.; Rosling, A. Archaeorhizomyces borealis sp. nov. and a sequence-based classification of related soil fungal species. *Fungal Biol.* **2014**, *118*, 943–955. https://doi.org/10.1016/j.funbio.2014.08.005

Molnar, O.; Schatzmayr, G.; Fuchs, E.; Prillinger, H. Trichosporon mycotoxinivorans sp. nov., a new yeast species useful in biological detoxification of various mycotoxins. *Syst. Appl. Microbiol.* **2004**, *27*, 661–671. https://doi.org/10.1078/0723202042369947

Ozimek, E.; Hanaka, A. Mortierella Species as the PlantGrowth-Promoting Fungi Present in the Agricultural Soils. *Agriculture* **2021**, *11*, 7. https://dx.doi.org/10.3390/agriculture11010007

Rodriguez, R.J.; White Jr, J.F.; Arnold, A.E.; Redman, R.S. Fungal endophytes: diversity and functional roles. *New Phytol.* **2009**, *182*, 314–330. https://doi.org/10.1111/j.1469-8137.2009.02773.x

Parveen, S.; Ali, M.I.; Aslam, M.; Ali, I.; Jamal, A.; Al-Ansari, M.M.; Al-Humaid, L.; Urynowicz, M.; Huang, Z. Optimizing biocatalytic potential of *Dipodascus australiensis* M-2 for degrading lignin under laboratory conditions. *Microbiol. Res.* **2022**, *265*, 127179; ISSN 0944-5013. https://doi.org/10.1016/j.micres.2022.127179.

Schlegel, M.; Münsterkötter, M.; Güldener, U.; Bruggmann, R.; Duò, A.; Hainaut, M.; Henrissat, B.; Sieber, C.M.; Hoffmeister, D.; Grünig, C.R. Globally distributed root endophyte *Phialocephala subalpina* links pathogenic and saprophytic lifestyles. *BMC Genom.* **2016**, *17*, 1015. https://doi.org/10.1186/s12864-016-3369-8

Špetík, M.; Berraf-Tebbal, A.; Pokluda, R.; Eichmeier, A. Pyrenochaetopsis kuksensis (Pyrenochaetopsidaceae), a new species associated with an ornamental boxwood in the Czech Republic. *Phytotaxa* **2021**, *498*, 177–185.

Vekiru, E.; Hametner, C.; Mitterbauer, R.; Rechthaler, J.; Adam, G.; Schatzmayr, G.; Krska, R.; Schuhmacher, R. Cleavage of zearalenone by Trichosporon mycotoxinivorans to a novel nonestrogenic metabolite. *Appl. Environ. Microbiol.* **2010**, *76*, 2353–2359.

Wang, H.M.D.; Cheng, Y.S.; Huang, C.H.; Huang, C.-W. Optimization of High Solids Dilute Acid Hydrolysis of Spent Coffee Ground at Mild Temperature for Enzymatic Saccharification and Microbial Oil Fermentation. *Appl. Biochem. Biotechnol.* **2016**, *180*, 753–765. https://doi.org/10.1007/s12010-016-2130-8

Weiss, M.; Bauer, R.; Sampaio, J.P.; Oberwinkler, F. *Systematics and Evolution*; McLaughlin, D.J., Spatafora,J.W. Eds.; The Mycota VII Part A; Springer-Verlag: Berlin/Heidelberg, Germany, 2014; pp. 331–355. https://doi.org/10.1007/978-3-642-55318-9_12

Commented [M1]: Please provide initials of editors.

Commented [WE2R1]: Done
